# Investigation of the relationship of sleep disorder occurring in fibromyalgia with central nervous system and pineal gland volume

**DOI:** 10.1017/neu.2024.49

**Published:** 2024-11-11

**Authors:** Fatih Çiçek, İlyas Uçar, Turgut Seber, Fatma Gül Ülkü Demir, Ali Türker Çiftçi

**Affiliations:** 1 Department of Anatomy, Faculty of Medicine, Institute of Health Sciences, Erciyes University, Kayseri, Türkiye; 2 Department of Anatomy, Faculty of Medicine, Erciyes University, Kayseri, Türkiye; 3 Department of Radiology, Kayseri City Education and Research Hospital, Kayseri, Türkiye; 4 Department of Physical Medicine and Rehabilitation, Kayseri City Hospital, Kayseri, Turkey; 5 Department of Biostatistics and Medical Information, Faculty of Medicine, Niğde Ömer Halisdemir University, Niğde, Türkiye

**Keywords:** Fibromiyalgia, sleep, tractography, pineal gland, spinoreticular tract

## Abstract

**Objective:**

Mechanisms of sleep disorders in fibromyalgia (FM) patients, such as insomnia, early morning awakenings and poor quality sleep, have not yet been proven and no consistent and effective treatment is yet available. The aim of this study was to investigate the pineal gland volume and the relationship between total fibre count, total fibre volume and total fibre length of the spinoreticular tract involved in regulation of sleep and wakefulness in terms of the mechanism of sleep disturbance.

**Methods:**

This study included only female cases, 31 with fibromyalgia and 31 controls. Pittsburgh Sleep Quality Index was used to assess sleep quality. Tractography of targeted pathway from brain diffusion MR images was calculated in Diffusion Studio Imaging (DSI) Studio programme and the volume of the pineal gland was calculated in ITK-SNAP programme.

**Results:**

The mean volume of the pineal gland was higher in control group (218.84 ± 64.45 mm^3^) than in fibromyalgia group (174.77 ± 48.65 mm^3^), which was statistically significant (*p* = 0.004). However, there was no statistically significant difference between two groups in total spinoreticular tract (TSRT), total volume (TSRTV), TSRT fractional anisotropy, TSRT mean diffusion, TSRT axial diffusion and TSRT radial diffusion of spinoreticular tract, which is involved in the regulation of sleep and wakefulness (*p* > 0.05).

**Conclusion:**

In conclusion, it is thought that the endocrine system may be more related to sleep disturbance in individuals with FM than central nervous system. Therefore, we believe that it may be more appropriate to work on the endocrine system rather than neural system in the treatment of sleep disturbance in patients with FM.

Significant outcomes

- It has been determined that the hormonal effect is greater than the central nervous system effect in the sleep disorder that occurs in fibromyalgia.
- There is a strong correlation between sleep disorder and pineal gland volume.
- In the treatment of the sleep disorder symptom in fibromyalgia, hormonal therapy should be used instead of neurological therapy.

Limitations

- To obtain a stronger association between sleep disturbance and pineal gland volume, pineal gland volume should also be examined in other diseases.
- Other pathways besides the spinoreticular tract should be examined for the symptom of sleep disorder in fibromyalgia.
- Conducting the study in a larger sample size to obtain more strong results.
- The endocrine changes of the population included in the study were not analysed.
- Factors such as occupational life and lifestyle that may cause sleep disturbance in fibromyalgia have not been examined.

## Introduction

Fibromyalgia (FM) is a common disorder characterised by chronic widespread pain, sleep problems (including non-restorative sleep), physical fatigue and cognitive difficulties (Hauser *et al*., 2015; Kartaloğlu et al., 2023). Its prevalence has been reported to be between 0.5 and 5% in the general population and up to 15.7% in the clinic. It has been reported that FM is more common in women than men, with a female to male ratio of 7–9:1 (Chen & McKenzie-Brown, 2015). FM patients frequently report sleep disorders, including insomnia, early morning awakenings and poor quality sleep (Spaeth *et al*., 2011). For this reason, the American College of Rheumatology included symptoms such as restless waking, fatigue and insomnia in the 2010 diagnostic criteria for fibromyalgia (Roizenblatt *et al*., 2011). In this context, it has been reported in the literature that 80% of the FM population has poor sleep quality. It has been reported that this may be related to the severity of FM symptomsv (Leon-LIamas *et al*., 2022). In this regard, it was mentioned that consistent and effective treatment is not yet available as the mechanisms potentially linking chronic widespread pain, sleep changes and mood disorders in FM have not yet been proven (Spaeth *et al*., 2011; Sarzi-Puttini *et al*., 2020). Moreover, the association of FM with other rheumatic disorders, chronic viral infections and systemic diseases has been well documented in several studies (Häuser *et al.*, 2015).

Studies investigating the relationship between sleep disturbance and the pineal gland have reported a relationship between sleep disturbance and pineal gland volume (Bumb *et al*., 2014; Song, 2019). In humans, the pineal gland is a small (100–150 mg), highly vascularised and secretory neuroendocrine organ. It is located in the midline of the brain, outside the blood-brain barrier, and is connected to the roof of the third ventricle by a short stalk (Arendt & Ailinas, 2022). The main function of the pineal gland is to receive information from the environment about the current light-dark cycle through the cyclical production and secretion of melatonin at night (dark period) and thus to play a role in the regulation of sleep and wakefulness (Takahashi *et al*., 2020; Arendt & Aulinas, 2022). Due to its integration with the somatomotor and neuroendocrine systems, the central nervous system has an important function in the regulation of sleep and wakefulness by playing a role in regulating the interaction of a living being with the external environment (Zoccoli & Amici, 2020). The ascending reticular activating system (ARAS) is a key component that regulates the overall activity of the cerebral cortex, including consciousness, sleep and arousal, through interactions with the reticular formation and various nuclei. The spinoreticular tract is responsible for part of the ARAS. Spinoreticular tract ascends along the lateral funiculus and terminates in the reticular formation (Kang & Im, 2023).

The aim of this study is to examine the volume of the pineal gland in terms of the mechanism of sleep disturbance between the patient group with FM and the control group and to investigate the relationship between the total number of fibres, total fibre volume, total fibre length of the spinoreticular tract, which is involved in the regulation of sleep and wakefulness, which will be the first in the literature.

## Materials and methods

This is a single-center, randomised controlled, prospective and cross-sectional cohort study conducted in the Physical Therapy and Rehabilitation Department of Kayseri City Hospital in accordance with the principles of the Declaration of Helsinki. Written informed consent was obtained from each patient and/or legal guardian while obtaining the images to be used in the study. In addition, approval for this study was obtained from the Nigde Omer Halisdemir University Non-Interventional Clinical Research Ethics Committee with protocol number 2023/115.

### Determination of sample size and study groups

To determine the sample size of the study, it was planned to include at least 31 female fibromyalgia patients and at least 31 female control group individuals with an effect size of 0.80, 90% power and 5% Type I error. This calculation was made with the G*power 3.1.9.6 programme. In this context, a total of 62 individuals, including 31 fibromyalgia patients and 31 control group, belonging to individuals whose MR images were taken between November 2023 and December 2023, were included in this study. The inclusion and exclusion criteria for this study are shown in Table 1 (Table 1). Since the incidence of fibromyalgia is significantly higher in favour of the female gender and in order to create optimum homogenisation, only female cases were included in both the patient and control groups, and the average age of the individuals participating in the study was 45.29 (Age range: 18–85).

**Table 1.**
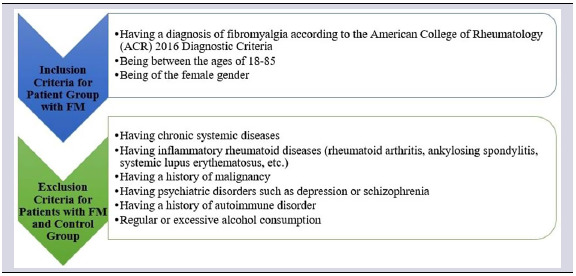
Inclusion and exclusion criteria of the individuals participating in the study

### Pittsburgh sleep quality ındex (PSQI)

Pittsburgh Sleep Quality Index (PSQI) was used to assess sleep quality in fibromyalgia and control groups. The PSQI is a scale that provides information about sleep quality and the type and severity of sleep disturbance in the last month. In the scale consisting of a total of 24 questions, 19 questions are answered by the respondent and 5 questions are filled in by the respondent’s bed-mate. The questions answered by the person are included in the evaluation, while the questions answered by the bed-mate are not included in the evaluation. Subjective sleep quality, sleep latency, sleep duration, habitual sleep efficiency, sleep disturbance, sleep medication use, and daytime dysfunction are assessed in 7 sub-dimensions with 19 questions answered by the respondent. Each item in the scale takes a value between 0 (no distress) and 3 (severe distress). The sum of the scores related to the seven sub-dimensions gives the total PSQI score. The score of each sub-dimension varies between 0 and 3. The total PSQI score ranged between 0 and 21. Those with a total score of 5 or less are considered to have ‘good’ sleep quality (Buysse *et al*., 1989; Buysse *et al*., 1991; Leon-LIamas *et al*., 2022). The Turkish validity and reliability study of the scale was conducted by Ağargün et al., and the internal consistency coefficient was reported as 0.80 (Agargun *et al*., 1996).

### Radiological evaluation

#### Tractography analysis

**Figure 1.**
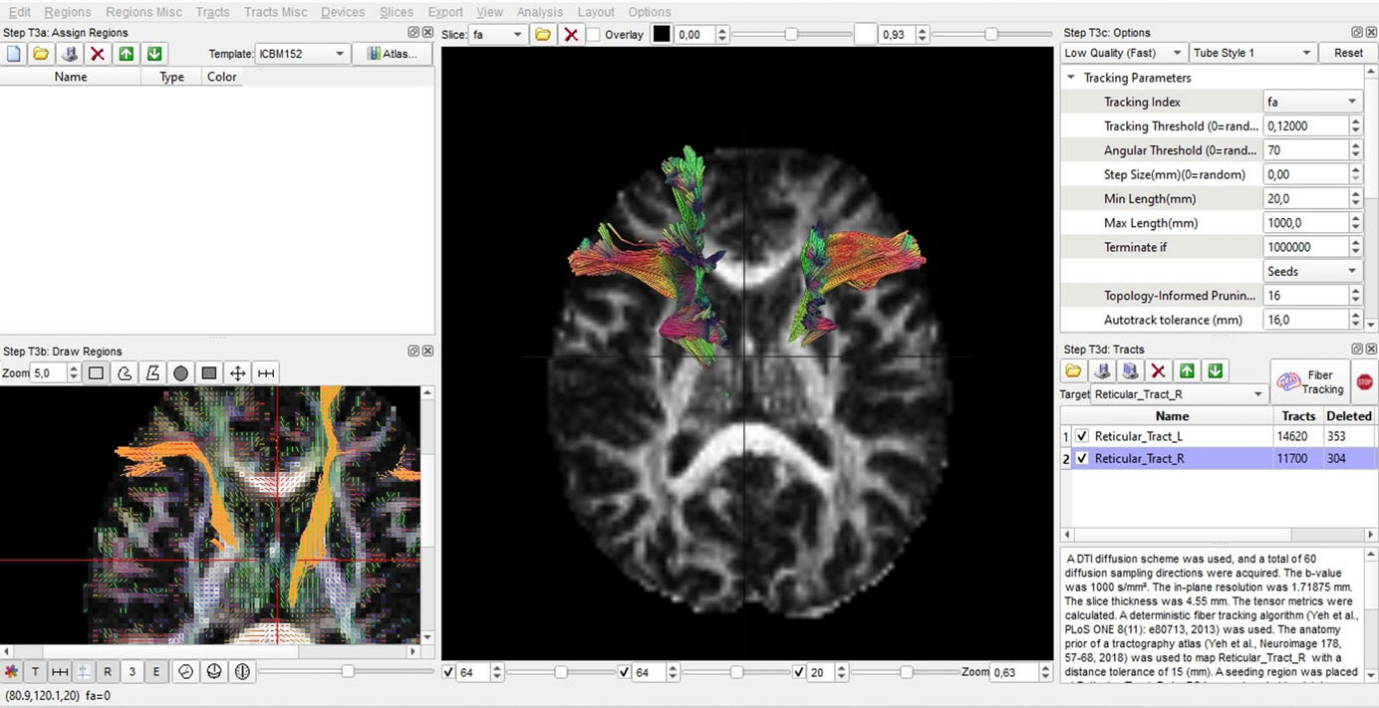
Tractography analysis of the spinoreticular tract in DSI Studio programme.

Tractography of the targeted tracts from brain diffusion MR images was performed in the Diffusion Studio Imaging (DSI) Studio programme downloaded from http://dsi-studio.labsolver.org/. Before starting the tractography process, in the ‘Fiber tracking’ tab; ‘Threshold’ was set to 0.20, ‘Angular Threshold’ was set to 70 degrees, ’Smoothing’ was set to 0.50, the shortest tract was set to 10 mm, the longest tract was set to 1000 mm, and ‘terminateif’ was set to 100,000 fibres. For each new image, these parameters were adjusted before starting the tractography procedure. Specifically, bilateral spinoreticular tract and all tracts in the brain were examined in our study (Fig. 1). The total number of fibres, average fibre length (in millimetres), average fibre volume (in cubic millimetres), the ratio of the number of fibres in the whole brain of the same individual to the total number of fibres in spinoreticular tract (Fiber ratio = number of fibres of the tracts in the whole brain / number of fibres in spinoreticular tract), fractional anisotropy (FA), mean diffusion (MD), axial diffusion (AD) and radial diffusion (RD) values of these tracts were calculated in tractography (Payas *et al*., 2023).

#### Pineal gland volume analysis

**Figure 2.**
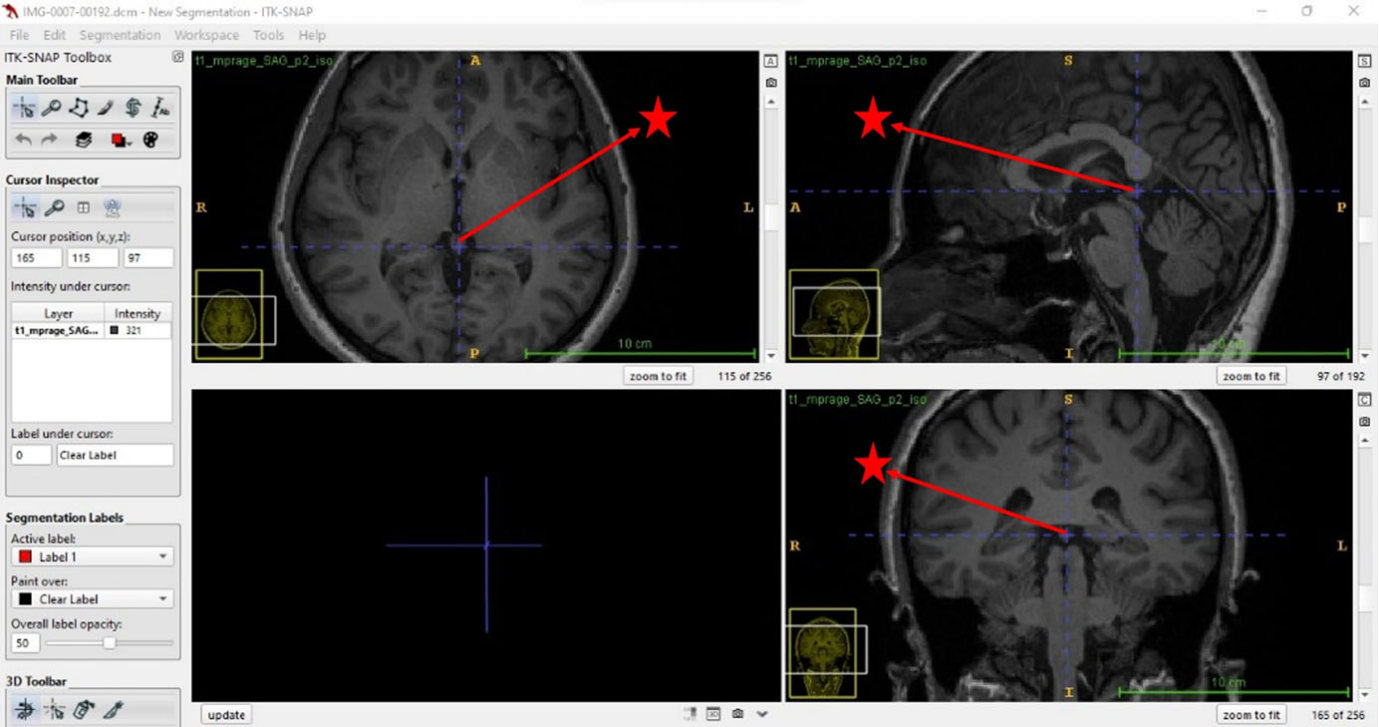
Identification of the pineal gland according to anatomical landmarks in T1-weighted magnetisation prepared with magnetisation-prepared gradient echo with rapid acquisition sequence (Red star: Pineal Gland).

Radiologic images used to calculate the volume of the pineal gland were obtained with a Siemens Magnetom Skyra, Netherlands 3 T (Tesla) MRI device. A T1-weighted magnetisation-prepared gradient echo with rapid acquisition (MPRAGE) sequence was used to image the anatomical structures of the brain. Corpus callosum, Colliculus superior, posterior part of Ventriculus tertius and Cisterna quadrigeminalis were mentioned as anatomical landmarks to identify the pineal gland (Fig. 2). T1-weighted MPRAGE sequence: sagittal, repetition time (TR) = 2300 ms, echo time (TE) = 3.4 ms, fip angle = 9°, FOV = 250 × 250 mm^2^, voxel size = 1 × 1 × 1 × 1, matrix = 256 × 256, slice thickness = 1 mm.

**Figure 3.**
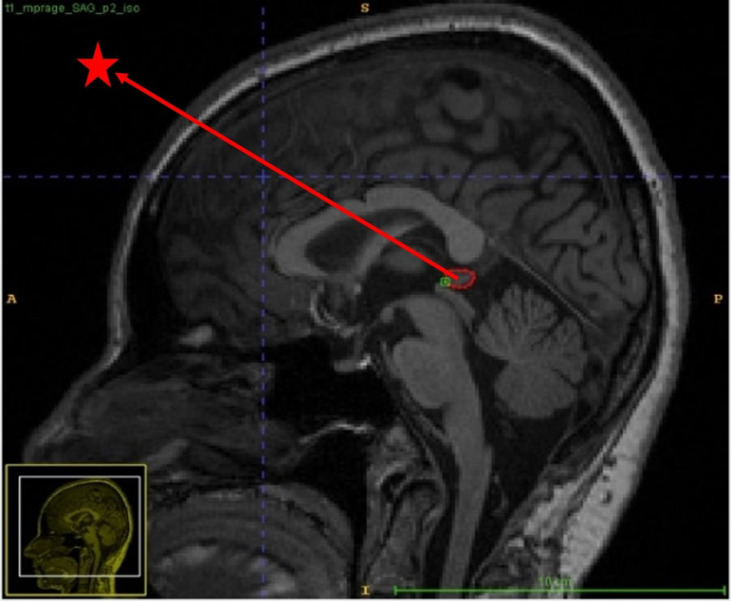
Segmentation of the pineal gland with the ITK-SNAP programme (Red star: Pineal Gland).

**Figure 4.**
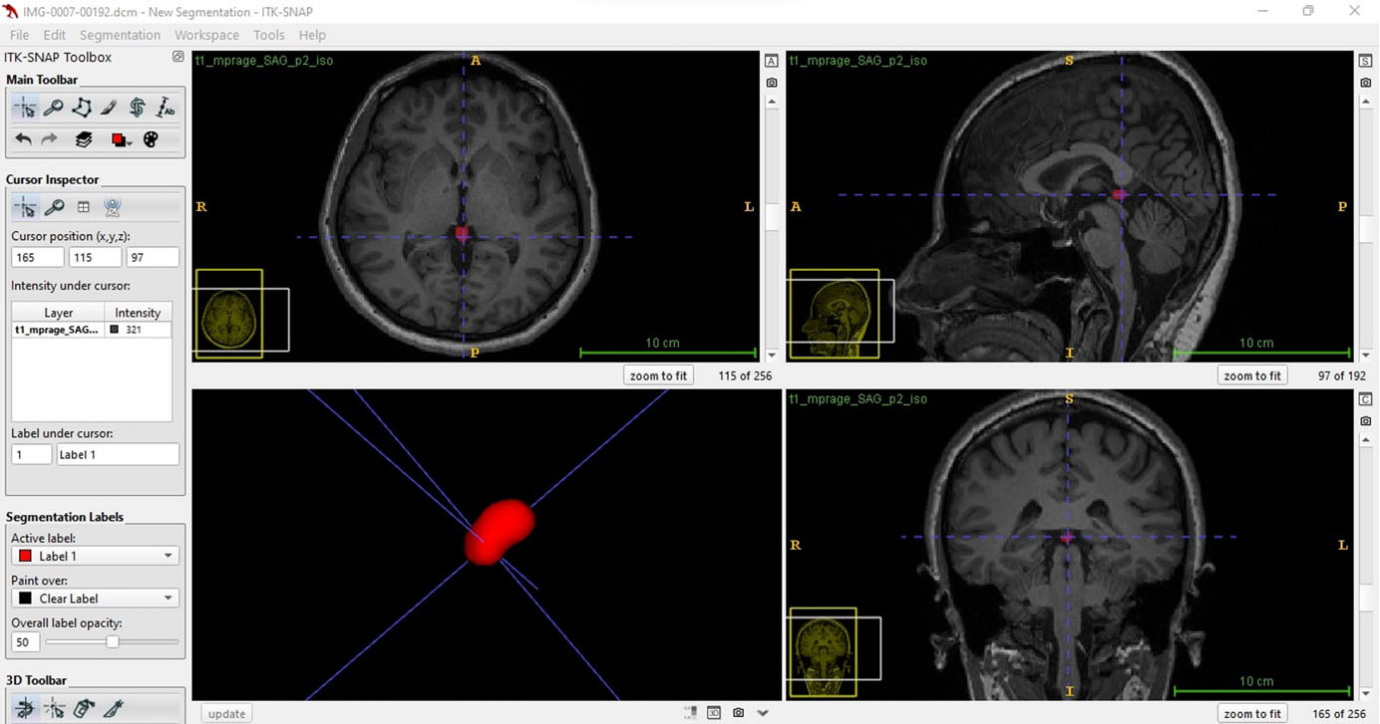
Calculation of pineal gland volume in mm^3^ and 3D reconstruction.

Insight Segmentation and Registration Tool Kit (ITK-SNAP) programme was used for pineal segmentation in this study. The ITK-SNAP programme has two different modes for segmentation, manual and semi-automatic (Fig. 3). In our study, the pineal gland volume was calculated using a semi-automated segmentation algorithm in ITK-SNAP. Segmentation was performed by loading MRI data obtained in Digital Imaging and Communications in Medicine format into the ITK-SNAP toolkit. After the images were loaded into the ITK-SNAP programme, ‘Active Label’ and ‘Draw-Over’ settings were made in the ‘Quick Label Picker’ tab, ‘Interpolate Labels’ option under the ‘Tool’ tab was selected and the volume of the pineal gland in the sagittal plane was calculated. This algorithm classifies pixels corresponding to the volume of the pineal gland in all MRI data slices and calculates the volume in mm^3^ (Fig. 4) (Batın et al., 2023).

### Statistical analysis

The Shapiro Wilk test was used to determine whether the variables were normally distributed and the measured parameters were found to be normally distributed. Numerical variables were summarised as Mean±S.Deviation and Min-Max. Independent *t*-test was used for two group comparisons. Pearson correlation was used to examine the relationship between numerical variables. Statistical analyses were performed with IBM SPSS version 22 (SPSS, Inc., Chicago, IL, USA). *p* < 0.05 was accepted as the level of statistical significance.

## Results

Table 2 shows the mean±standard deviation and min-max values of the parameters measured.

**Table 2.** Descriptive statistics values of the measured parameters

Variables	N	Mean±SD	Min-Max
Age (Years)	62	45.29 ± 9.04	24.00–68.00
BMI (kg/m^2^)	62	27.32 ± 4.10	17.41–35.38
PSQI	62	12.98 ± 3.67	7.00–21.00
PGV (mm^3^)	62	197.16 ± 60.94	91.00–334.00
TTR	62	72602.62 ± 1664.26	67254.00–77150.00
TTR/TSRT	62	50.14 ± 18.12	19.04–88.26
TSRT	62	1657.95 ± 643.81	815.00–3819.00
TTRV (mm^3^)	62	806415.03 ± 60387.71	690511.00–976584.00
TSRTV (mm^3^)	62	26580.43 ± 4243.23	18631.13–36699.40
TSRTL (mm)	62	255.56 ± 12.65	226.73–285.69
TSRT FA	62	0.44 ± 0.02	0.41–0.48
TSRT MD	62	0.85 ± 0.05	0.78–0.96
TSRT AD	62	1.29 ± 0.05	1.19–1.40
TSRT RD	62	0.63 ± 0.05	0.56–0.74

(**BMI(kg/m**^**2**^ **):** Body mass index; **PSQI:** Pittsburgh sleep quality index; **PGV(mm**^**3)**^: Pineal gland volume; **TTR**: Total tracts; **TSRT:** Total spinoreticular tracts**; TTR/TSRT:** Total tracts/Total spinoreticular tracts**; TTRV(mm**^**3)**^: Total tracts volume**; TSRTV:** Total spinoreticular tracts volume**; TSRTL:** Total spinoreticular tracts length**; TSRT FA:** FA of the total spinoreticular tracts**; TSRT MD:** MD of the total spinoreticular tracts**; TSRT AD:** AD of the total spinoreticular tracts**; TSRT RD:** RD of the total spinoreticular tracts).

Table 3 shows the comparison of the measured parameters between fibromyalgia and control group. Accordingly, Table 3 shows that there is no statistically significant difference between the two groups in terms of body mass index (BMI) (kg/m^2^) and age (*p* = 0.204). This supports the homogeneous distribution of both groups with gender. Table 3 shows that the mean volume of the pineal gland was higher in the control group (218.84 ± 64.45 mm^3^) than in the fibromyalgia group (174.77 ± 48.65 mm^3^), which is statistically significant (*p* = 0.004). This proves that sleep disturbance in FM is related to pineal gland volume. In addition, when the PSQI value given in Table 3 is examined, it is seen that there is a statistically significant difference between fibromyalgia (14.80 ± 3.03) and control group (4.17 ± 2.65) (*p* < 0.001)(Table 3). This supports that the pineal gland regulates sleep and wakefulness by secreting melatonin, which is why fibromyalgia patients have sleep disturbance symptoms. However, there was no statistically significant difference between the two groups in total spinoreticular tracts count (TSRT), total volume (TSRTV), FA (TSRT FA), MD (TSRT MD), AD (TSRT AD) and RD (TSRT RD) of spinoreticular tract, which is involved in the regulation of sleep and wakefulness (*p* > 0.05) (Table 3). These findings suggest that sleep disturbance in FM is not related to the central nervous system.

**Table 3.** Comparison of parameters between fibromyalgia and control group

Variables	Participants with FM (N = 31)	Control Group (N = 31)	*p*
	Mean±SD	Mean±SD	
**Age (Years)**	46.80 ± 8.01	43.84 ± 9.86	0.204
**BMI (kg/m**^**2**^ **)**	28.23 ± 3.99	26.43 ± 4.07	0.088
**PSQI**	14.80 ± 3.03	4.17 ± 2.65	**<0.001**
**PGV (mm**^**3**^ **)**	174.77 ± 48.65	218.84 ± 64.45	**0.004**
**TTR**	72605.47 ± 2157.16	72599.87 ± 1020.41	0.990
**TTR/TSRT**	49.16 ± 18.62	51.09 ± 17.88	0.681
**TSRT**	1704.57 ± 664.50	1612.84 ± 630.77	0.582
**TTRV (mm**^**3**^ **)**	810297.50 ± 69312.59	802657.81 ± 51181.99	0.625
**TSRTV (mm**^**3**^ **)**	26579.53 ± 4062.28	26581.30 ± 4478.61	0.994
**TSRTL (mm)**	253.72 ± 14.94	257.34 ± 9.88	0.267
**TSRT FA**	0.44 ± 0.02	0.45 ± 0.01	0.241
**TSRT MD**	0.86 ± 0.05	0.85 ± 0.04	0.885
**TSRT AD**	1.28 ± 0.05	1.29 ± 0.04	0.600
**TSRT RD**	0.64 ± 0.05	0.63 ± 0.04	0.661

Independent sample *t*-test was used for group comparison.

(**BMI(kg/m**^**2**^ **):** Body mass index; **PSQI:** Pittsburgh sleep quality index; **PGV(mm**^**3)**^: Pineal gland volume; **TTR**: Total tracts; **TSRT:** Total spinoreticular tracts**; TTR/TSRT:** Total tracts/Total spinoreticular tracts**; TTRV(mm**^**3)**^: Total tracts volume**; TSRTV:** Total spinoreticular tracts volume**; TSRTL:** Total spinoreticular tracts length**; TSRT FA:** FA of the total spinoreticular tracts**; TSRT MD:** MD of the total spinoreticular tracts**; TSRT AD:** AD of the total spinoreticular tracts**; TSRT RD:** RD of the total spinoreticular tracts).

Table 4 shows the correlation between the parameters measured in the fibromyalgia group and Table 5 shows the correlation between the parameters measured in the control group. Accordingly, both Table 4 and Table 5 show that there is no statistically significant relationship between PGV and TTR/TSRT, TSRT, TSRTV, TSRTL parameters (*p* > 0.05). However, in the fibromyalgia group, there was a moderate positive linear relationship between PGV and TSRT AD parameters (*p* = 0.027; *r* = 0.403). In addition, the correlation between TTR and TSRT parameters in the fibromyalgia group was not statistically significant (*p* = 0.185) (Table 4). However, a statistically significant negative correlation was found between TTR and TSRT parameters in the control group (*p* = 0.018; r= −0.424) (Table 5).

**Table 4.** Correlation table of the parameters measured in the fibromyalgia group

		BMI	PGV	TTR	TTR/TSRT	TSRT	TSRTV	TSRTL
**PSQI**	r	−0.196	−0.164	−0.077	−0.018	−0.057	0.001	0.050
	p	0.299	0.387	0.686	0.923	0.764	0.998	0.794
	N	31	31	31	31	31	31	31
**AGE**	r	0.439*	0.106	−0.245	0.009	0.003	−0.230	−0.210
	p	**0.015**	0.578	0.193	0.962	0.986	0.222	0.265
	N	31	31	31	31	31	31	31
**BMI**	r	1	0.382*	0.107	0.187	−0.189	−0.024	0.208
	p		**0.037**	0.575	0.323	0.318	0.900	0.270
	N		31	31	31	31	31	31
**PGV**	r		1	0.098	0.134	−0.087	−0.069	0.324
	p			0.605	0.481	0.649	0.717	0.080
	N			31	31	31	31	31
**TTR**	r			1	0.215	−0.249	0.207	0.372*
	p				0.253	0.185	0.273	**0.043**
	N				31	31	31	31
**TTR/TSRT**	r				1	−0.935**	−0.455*	0.048
	p					**<0.001**	**0.012**	0.802
	N					31	31	31
**TSRT**	r					1	0.415*	0.041
	p						**0.023**	0.831
	N						31	31
**TSRTV**	r						1	0.228
	p							0.226
	N							31

* Correlation is significant at the 0.05 level (2-tailed).

** Correlation is significant at the 0.01 level (2-tailed).

(**BMI(kg/m**^**2**^: Body mass index; **PSQI:** Pittsburgh sleep quality index; **PGV(mm**^**3)**^: Pineal gland volume; **TTR**: Total tracts; **TSRT:** Total spinoreticular tracts**; TTR/TSRT:** Total tracts/Total spinoreticular tracts**; TTRV(mm**^**3)**^: Total tracts volume**; TSRTV:** Total spinoreticular tracts volume**; TSRTL:** Total spinoreticular tracts length**; TSRT FA:** FA of the total spinoreticular tracts**; TSRT MD:** MD of the total spinoreticular tracts**; TSRT AD:** AD of the total spinoreticular tracts**; TSRT RD:** RD of the total spinoreticular tracts).

**Table 5.** Correlation table of the parameters measured in the control group

		BMI	PGV	TTR	TTR/TSRT	TSRT	TSRTV	TSRTL
**PSQI**	r	−0.096	0.100	−0.227	−0.168	0.283	0.060	0.228
	p	0,614	0.599	0.228	0.374	0.129	0.754	0.226
	N	31	31	31	31	31	31	31
**AGE**	r	0.680**	0.111	−0.175	0.110	−0.138	−0.084	−0.248
	p	**<0.001**	0.552	0.348	0.556	0.460	0.651	0.178
	N	31	31	31	31	31	31	31
**BMI**	r	1	0.221	−0.391*	−0.008	−0.084	0.107	−0.170
	p		0.233	**0.030**	0.964	0.655	0.567	0.361
	N		31	31	31	31	31	31
**PGV**	r		1	−0.155	0.013	−0.049	−0.035	−0.283
	p			0.404	0.946	0.793	0.851	0.123
	N			31	31	31	31	31
**TTR**	r			1	0.496*	−0.424*	−0.359*	0.085
	p				**0.005**	**0.018**	**0.048**	0.650
	N				31	31	31	31
**TTR/TSRT**	r				1	−0.897**	−0.601**	−0.264
	p					**<0.001**	**<0.001**	0.151
	N					31	31	31
**TSRT**	r					1	0.641**	0.478*
	p						**<0.001**	**0.007**
	N						31	31
**TSRTV**	r						1	0.482*
	p							**0.006**
	N							31

* Correlation is significant at the 0.05 level (2-tailed).

** Correlation is significant at the 0.01 level (2-tailed).

(**BMI(kg/m**^**2**^: Body mass index; **PSQI:** Pittsburgh sleep quality index; **PGV(mm**^**3)**^: Pineal gland volume; **TTR**: Total tracts; **TSRT:** Total spinoreticular tracts**; TTR/TSRT:** Total tracts/Total spinoreticular tracts**; TTRV(mm**^**3)**^: Total tracts volume**; TSRTV:** Total spinoreticular tracts volume**; TSRTL:** Total spinoreticular tracts length**; TSRT FA:** FA of the total spinoreticular tracts**; TSRT MD:** MD of the total spinoreticular tracts**; TSRT AD:** AD of the total spinoreticular tracts**; TSRT RD:** RD of the total spinoreticular tracts**).**

## Discussion

In this study, the volume of the pineal gland, which is responsible for melatonin secretion, was evaluated between individuals with FM and healthy control group in order to investigate the mechanism of symptoms related to sleep disturbance in individuals with FM. In addition, total fibre number, fibre length and total fibre volume of spinoreticular tract, which has an important function in the regulation of sleep and wakefulness, were examined for the first time between the two groups in this study. Sleep quality of both groups was determined with the PSQI scale. When the total PSQI value was compared between the groups, it was found to be statistically significant that the fibromyalgia group had worse sleep quality than the control group (*p* < 0.001).

Karabas et al., examined the volume of the epiphyseal gland in 26 FM patients (23 females, 3 males) with a mean age of 40.62 years and 26 controls (23 females, 3 males) with a mean age of 37.42 years. They stated that there was no statistically significant difference between the patient and control groups in terms of PGV (*p* = 0.374) (Karabas *et al*., 2022). Leon-LIamas et al., used the PSQI scale to assess sleep quality in the FM group but did not use any scale for the control group in their study of 30 FM patients with a mean age of 53 years and 20 controls. They then examined the volume of the pineal gland between both groups and found that there was no statistically significant difference (*p* = 0.426) (Leon-LIamas *et al*., 2022). In this study, FM and control groups were analysed in terms of the volume of the pineal gland and it was found to be statistically significant (*p* = 0.004) that the control group (218.84 ± 64.45) had a higher mean pineal gland volume than the FM group (174.77 ± 48.65) (Table 3). The reason why the result of this study is different from the other studies is that in the other studies, only the sleep quality of the FM group was assessed with PSQI, while in this study, the sleep quality of both groups was assessed with PSQI scale and statistically significant results were obtained between both groups. Because it should be taken into consideration that the individuals in the control group will also have poor sleep quality due to other reasons. Thus, we believe that healthier and more accurate results will be obtained.

Furthermore, Takahashi et al., reported a relationship between the parenchymal volume of the pineal gland and the level of melatonin secreted. This supports that sleep disturbance in the FM group is associated with lower pineal gland volume compared to the control group. We believe that these results should be supported by clinical studies examining the relationship between pineal gland volume and endocrine changes to increase the reliability of these results (Takahashi *et al*., 2020).

The reticular formation of the brainstem is directly or indirectly connected to almost all levels of the central nervous system and performs a variety of essential functions required for basic life activities such as motor control, wakefulness, sleep and -wakefulness cycle, pain perception and visceral activity. The reticular formation is primitive and scattered. The more primitive the animal, the greater the proportion of reticular formation in the brainstem. Representative connections between the reticular formation and the spinal cord tract include the reticulospinal and spinoreticular tracts (Kiernan & Barr, 2009; Martins & Tavares, 2017).

In this study, total fibre number, fibre length, total volume, FA, MD, RD and AD values of spinoreticular tract, which is the first in the literature, were examined to investigate the relationship between central nervous system and sleep disturbance in patients with FM. There was no statistically significant difference between the FM group and the healthy control group regarding total fibre number, fibre length, total volume, FA, MD, RD and AD values of spinoreticular tract (*p* > 0.05). In addition, when the total fibre count of all tracts in the brain was examined in both groups, no statistically significant difference was found between the two groups (*p* > 0.05). With these results, we believe that sleep disturbance in patients with FM may be related to the endocrine system rather than the neural system.

## Conclusion

In this study, the relationship between neural and endocrine systems and sleep disturbance in individuals with FM was examined. As a result, it is thought that the endocrine system may be more related to sleep disturbance in individuals with FM than the neural system. Therefore, we believe that it may be more appropriate to work on the endocrine system rather than the neural system in the treatment of sleep disturbance in patients with FM. In addition, it should be examined prospectively whether the reticulospinal tract is also associated with sleep disturbance in other diseases.
